# A Novel Autonomous Perceptron Model for Pattern Classification Applications

**DOI:** 10.3390/e21080763

**Published:** 2019-08-06

**Authors:** Alaa Sagheer, Mohammed Zidan, Mohammed M. Abdelsamea

**Affiliations:** 1 College of Computer Science and Information Technology, King Faisal University, AlAhsa 31982, Saudi Arabia; 2 Center for Artificial Intelligence and Robotics (CAIRO), Faculty of Science, Aswan University, Aswan 81528, Egypt; 3 University of Science and Technology, Zewail City of Science and Technology, October Gardens, 6th of October City, Giza 12578, Egypt; 4 Department of Mathematics, Faculty of Science, Assiut University, Assiut 71515, Egypt; 5 School of Computer Science, University of Nottingham, Nottingham NG8 1BB, UK

**Keywords:** machine learning, pattern classification, artificial neural networks, quantum-inspired neural network, soft computing

## Abstract

Pattern classification represents a challenging problem in machine learning and data science research domains, especially when there is a limited availability of training samples. In recent years, artificial neural network (ANN) algorithms have demonstrated astonishing performance when compared to traditional generative and discriminative classification algorithms. However, due to the complexity of classical ANN architectures, ANNs are sometimes incapable of providing efficient solutions when addressing complex distribution problems. Motivated by the mathematical definition of a quantum bit (qubit), we propose a novel autonomous perceptron model (APM) that can solve the problem of the architecture complexity of traditional ANNs. APM is a nonlinear classification model that has a simple and fixed architecture inspired by the computational superposition power of the qubit. The proposed perceptron is able to construct the activation operators autonomously after a limited number of iterations. Several experiments using various datasets are conducted, where all the empirical results show the superiority of the proposed model as a classifier in terms of accuracy and computational time when it is compared with baseline classification models.

## 1. Introduction

Classification is one of the most active research areas in the machine learning domain and plays a significant role in many applications such as product inspection, quality control, fault detection, medical diagnosis, credit scoring, bankruptcy prediction and speech recognition, to mention a few [1]. Pattern classification models can be categorized into two broad classes: parametric and non-parametric.

Parametric models such as the support vector machine (SVM) [2] and decision tree [3] rely on the hypothesis that the training observations should be plentiful and obey a certain distribution. This provides accurate outcomes, but also restricts their scope. Likewise, discriminant analysis models [4] have been designed mainly based on the Bayesian decision theory. In such models, the underlying model probability should be estimated in order to provide the posterior probabilities upon which the classification decisions are made [4,5]. The major restriction of this class of models is that the underlying statistical assumptions should be satisfied to provide accurate classification boundaries. Therefore, prior knowledge about model capabilities and data properties should be considered when building a model [1].

On the other hand, non-parametric models such as the artificial neural network (ANN) can provide robust solutions to solve complex real-world classification problems with no statistical assumptions about the distribution of the data. However, there are some restrictions to the use of non-parametric models. In this paper, we use ANN as an example to shed light on the limitations of non-parametric models and to motivate our solution. For example, the scope of ANN is limited to the availability of a large number of training observations, which requires too many hidden nodes and therefore excessive training time and computing requirements [6]. Furthermore, training an ANN requires the use of an adaptive method to determine a suitable network structure and an iterative update for connection weights, which are, in turn, computationally expensive [7].

The expensive computation of ANNs, the difficulty of fine-tuning their hyper-parameters and the identification of an optimal network structure have motivated research groups to investigate novel approaches to overcome these limitations. One of these approaches was to integrate other learning techniques with ANNs to enhance their overall computational complexity. Examples of these techniques are fuzzy logic [8], genetic algorithms [9], and evolutionary computation [6].

Recently, there have been unremitting research efforts to adopt quantum computation into machine learning and artificial intelligence contexts [10]. This research trend deals with the capability of quantum computation applied to neural computation, capitalizing on the superposition power of the quantum bit (qubit), which is different from its classical counterpart (bit). Quantum computing-based neural networks and quantum-inspired neural networks (QiNNs) [11] have demonstrated better performance over classical ANNs in terms of effectiveness and efficiency [12,13,14,15,16,17,18,19,20,21]. QiNN models can be further divided into two main categories: QiNN models that are only implemented on quantum computers, which strive to break out of labs [22], and models that take advantage of both QiNN and ANN and could be implemented on classical computers [11,12,14,15,16]. These models are the main focus of this paper.

Most of the previously-proposed QiNN approaches were designed mainly to improve the robustness of classical perceptron models [23] using the computational power of the qubit in the selection of the perceptron’s activation operator [16,20,24,25]. However, some of these approaches are computationally very expensive, especially when they are implemented on classical computers. On the other hand, some of these approaches create a new structure of a quantum neuron [16,24,25], which is not compatible with the quantum computing postulates [26]. In addition, such quantum neurons are still sensitive to the selection of appropriate activation operators. In this paper, we propose a novel autonomous perceptron model (APM) inspired by the computational power of the qubit. The proposed model is capable of achieving efficient pattern classification experiment results using the classical computer only. Accordingly, the main contributions of the proposed model can be summarized as follows:

- The APM is designed with an optimal neural structure of only one single neuron to classify nonlinear separable datasets.
- The APM is able to construct the neural network activation operators autonomously.
- The APM is a robust classifier that is able to compete favourably with several standard classifiers and can be implemented in a limited number of iterations.

Here, the empirical experiments show that the proposed perceptron model outperforms other counterpart models presented in [16,20] when learning the logical XOR function, which cannot be implemented by the classical perceptron. Moreover, the proposed APM model outperforms several baseline linear and nonlinear classification models, such as multilayer perceptron, linear discriminate analysis, SVM and AdaBoost; in terms of accuracy and computational time of classification problems using several real benchmark datasets.

The rest of the paper is organized as follows: Section 2 shows a few basic concepts of quantum computation and the classical perceptron. A comprehensive overview of related work is provided in Section 3, along with the limitations of previous works. Section 4 presents the proposed APM model and its learning settings. Section 5 provides the computational capability of the proposed model. Section 6 shows the experimental results of the proposed model in learning various classification problems using real and synthetic datasets. A time complexity analysis of the APM is reported in Section 7. Section 8 gives a thorough discussion of the results of the paper. Eventually, Section 9 concludes the paper and its findings.

## 2. Background

This section shows an overview of a few basic concepts needed for the paper. Readers who are familiar with these concepts may skip this section.

### 2.1. Quantum Computation

Quantum computation has attracted much attention in the last two decades after the development of a quantum algorithm that was able to factorize large integers in polynomial time [27]. Generally speaking, quantum computation aims to develop computer technology based on the postulates of quantum mechanics [26]. Classical physics applies to things that human beings can see, whereas quantum physics applies to things that are at the scale of atoms or below. Quantum computation essentially capitalizes on two properties of quantum particles followed by the postulates of quantum mechanics: (i) superposition and (ii) entanglement [26]. Superposition is a one-particle property, while entanglement is a characteristic of two or more particles. The computer that uses postulates of quantum mechanics and performs the computation is called a quantum computer [28].

### 2.2. Quantum Bit

The quantum computer is completely different from the digital/classical computer [28]. For instance, in the classical computer, information is stored and transformed using the binary system as bits; whereas in the quantum computer, information is stored and transformed as quantum bits (qubits). Unlike the classical bit, which is represented as either zero or one, a qubit is represented by a linear combination, often called a superposition, of the two base states |0〉 and |1〉, as follows:

(1) $$|\psi \rangle =a|0\rangle +b|1\rangle =\left(\begin{array}{c} a\\ b \end{array}\right)$$

Here, *a* and *b* are complex numbers, usually called the probability amplitudes, and therefore, they satisfy ${\left|a\right|}^{2}+{\left|b\right|}^{2}=1$. In this representation, we used Dirac’s notations “ket” |.〉 and “bra” 〈.|.

### 2.3. The Perceptron Model

The single-perceptron model can be described as a single neuron with a sgn (i.e., threshold) activation function, which can be used to solve simple binary classification problems [29]. More precisely, it can be illustrated using the following equation:

(2) $$f\left(x\right)=\left\{\begin{array}{c} 1\;\;if\;\;w.x+B>0\\ 0\;\;otherwise \end{array}\right.$$

where *w* is a weight vector, *x* is the input, and *B* is a bias. The output value f(x) is then used to classify an input pattern *x* based on learned weights. The perceptron has the ability to determine a separating hyperplane if the patterns are linearly separable in finite steps; otherwise, a convergence issue occurs [30].

## 3. Related Work

This section provides a comprehensive review for previous works along with their limitations.

### 3.1. Previously Proposed Models

ANNs are the most widespread type of computational algorithm inspired by the function and architecture of the biological neural networks in the human brain [7]. Like a simplified brain model, an ANN can be represented as an *n*-dimensional graph where the nodes are called neurons and their connections are weighted by specific parameters [7]. They are nonlinear and non-parametric models that are easy to use and understand when compared with traditional statistical methods, which are parametric models. ANNs along with learning algorithms are widely used to solve various classification, recognition, and forecasting problems [1,7]. However, more than 30 years of research in ANNs has demonstrated that traditional ANNs are not capable of achieving low-cost learning due to their expensive computation requirements. Furthermore, in the current era of big data, the pressure to find innovative approaches to ANNs has been rising [31].

In recent years, there have been persistent research efforts to evoke quantum computation capabilities in ANN algorithms. Superposition is a property that aligns with the linearity of the system operators, allowing for a powerful form of parallel computation that is able to develop more efficient algorithms than the known classical algorithms [18]. The first attempt to use quantum computing in the classical realm was done by Grover et al. [32] who presented an algorithm to find an element in an unordered dataset quadratically faster than its classical counterpart. At that early time, it was reported that the computational power of the QiNN would be higher than that of ANN in terms of effectiveness and efficiency [33]. Currently, integrating quantum computation capabilities into ANNs is still an open and challenging trend of research [34].

One of the earlier contributions to this trend was given by Menneer et al. [35], who presented the first QiNN algorithm. Ventura and Martinez [17] proposed a quantum implementation of the associative memory model. Likewise, Narayanan and Menneer [36] showed that QiNN is not only more efficient, but also more powerful when compared with the traditional ANN. Moreover, Gupta and Zia [37] defined a novel QiNN computational model based on the concept of the Deutsch computational model. Next, Kouda et al. [38] proposed the qubit neural network through the use of some quantum characteristics such as quantum superposition and probability interpretation.

Most of the above-mentioned efforts relied on one of two approaches, either the classical feed forward neural networks (FFNNs) or the Hopfield networks [17]. FFNNs cannot be simulated by linear and unitary dynamics of quantum computation directly due to the nonlinear and dissipative dynamics of the classical neurons [34], whereas the Hopfield networks, which are mainly based on the associative memory rather than a nonlinear activation function, use the Hamming distance to find the closest unit to the input pattern. In fact, a few real-life applications rely on the Hamming distance, which thereby limits the usability of the Hopfield QiNN [34].

Another direction that has attracted much attention is based on the classical Rosenblatt’s perceptron model [23], and several attempts have been made to develop a quantum equivalent for this classical model. In principle, perceptron models, as linear classifiers, play an important role in the foundations of classical neural networks [39]. The first quantum perceptron model, which was considered as “network with a teacher”, was presented by Altaisky et al. [24]. Fei et al. [25] described a model for quantum perceptron and discussed its quantum learning rule, which was able to learn the XOR function successfully and had the same computational power as the two-layer perceptron. Zhou et al. [13,16] presented a quantum perceptron model built on the combination of the classical perceptron model and quantum computing. Zhou’s model has computing power that the traditional perceptron is unable to realize. Meng et al. [40] introduced a model based on a quantum computational multi-agent system with a reinforcement-learning approach in which each perceptron acts as a dynamic parallel computing agent to increase its speed, computational power, and learning ability.

Most of the previously proposed QiNNs follow supervised learning approaches. More recently, there has been motivation to develop QiNN without a teacher or self-organized networks, where weight factors are determined by the parameters of the problem to be solved [34]. The first attempt in this trend was proposed by Siomau [20], who developed an autonomous quantum analogue for the classical Rosenblatt’s perceptron, exploiting the superposition principle of quantum systems. Siomau’s model is able to learn the XOR logical function, to perform the classification on previously unseen classes, and to recognize the superpositions of the learned classes. All these tasks are unrealisable with a classical perceptron with a single neuron.

Next, several attempts were made in order to exploit the use of quantum mechanics in particular scenarios. For instance, Bhattacharyya et al. [41] proposed a quantum model inspired by backpropagation multilayer perceptron based on quantum gates for the prediction of the adsorption behaviour often exhibited by calcareous soil. Likewise, Schuld et al. [39] introduced a quantum perceptron model based on the quantum phase estimation algorithm that imitates the step-activation function of a classical perceptron. They employed it efficiently to construct more complex structures such as trainable quantum neural networks. Da Silva et al. [18] proposed a quantum perceptron over a field (QPF), based on quantum gates, as a direct generalization of a classical perceptron to solve some drawbacks found in previous models of the quantum perceptron. Chen et al. [34] presented the quantum probability neural network (QPNN) model, which uses the quantum parallelism property to trace all possible network states in order to improve the result and reduce the effect of the noise. In addition, QPNN can be used as memory to retrieve the most relevant data and even to generate new ones. the QPNN model has been applied to the Iris dataset and the Modified National Institute of Standards and Technology (MNIST) dataset using much fewer neuron resources to obtain a better result than the classical FFNN.

In 2017, Yamamoto et al. [42] introduced the Multidimensional Input Quantum Perceptron (MDIQP) using controlled changes of ancillary qubits in combination with phase estimation and learning algorithms. As an extension of the capabilities of the classical perceptron, MDIQP is capable of representing and classifying arbitrary sets of multidimensional data, either quantum or classical, which may not be linearly separable. Neto et al. [43] also introduced a quantum perceptron with an internal state memory that can be changed during neuron execution. Neto’s perceptron reproduces the step function of the inner product between input and weights and has a memory that can be updated during its own execution. Lastly, Liu et al. [44] presented a quantum perceptron algorithm based on unitary weights, where the singular value decomposition of the total weight matrix from the training dataset is retained in order to convert the weight matrix to be unitary. The model was validated using a number of universal quantum gates within one iteration.

### 3.2. Limitations and Motivation

Generally speaking, the QiNNs models described in Section 3.1 can be divided into two categories. First, some QiNNs models should be implemented on the quantum computer, such as the models described in [18,36]. It is known that universal quantum computers strive to break out of labs [22]. Even if the quantum computer is finally introduced, we do not know which hardware will be used [18,45]. The second category is QiNNs, which can be implemented on the classical computer, such as the models described in [11,12,14,15,16,38]. These QiNNs models are not real quantum models; rather, they are classical neural networks inspired by quantum computation. The proposed model in this paper falls into the latter QiNN approach.

Most of the QiNN approaches have been designed mainly to improve the robustness of the classical perceptron model [23] using the computational power of quantum computation in the selection of the activation operator of the perceptron. However, approaches such as [16,20,24,25] are still computationally very expensive when they are implemented on the classical computer. Moreover, each of the quantum perceptron approaches proposed in [16,24,25] suggested a new structure of the quantum neuron or perceptron. Those new structures of the quantum neuron are not compatible with the quantum computing postulates and are still sensitive to the selection of the appropriate activation operators [46]. Although, the model in [20] is an autonomous version of the quantum perceptron, it is a linear model and does not have the potential to learn nonlinear gates, such as the Z-gate and Hadamard-gate.

Recent models, such as the model described in [39], which presents a direct quantization of the classical neuron, have a few advantages in terms of quantum computation, but they can be used as a building block of other QiNNs in the future [34]. Furthermore, the model presented in [18] was a QiNN; however, the authors assumed that it could be implemented on the quantum computer, even though the perceptron model of [18] had no activation function and no experimental results were provided in the paper to verify its performance in real applications [34].

It is worth mentioning that the model described in [34] may be similar to the model described in our paper; however, we observed that the other model [34] was very sensitive, as the square error was adopted as a loss function for each sample for the sake of simplifying the problem for the model. In addition, we noticed that the model adopts the classical sampling strategy to achieve layer reduction. In fact, we believe that such a simple strategy can easily fall into the dilemma of local optima after exhaustive learning. Furthermore, a few parameters of the model are adjusted manually.

Although the perceptron model described in [42] can be used as a fundamental cell for the construction of more complex QiNN models, the model was not evaluated properly in real experiments or using real datasets. In addition, it uses a number of registers, and thereby qubits, proportional to the size of the input data, so it requires more computation resources if the size of the input dataset is large. Finally, there are two quantum perceptron models similar to the perceptron model proposed in this paper: First is the perceptron presented in [43], but the authors did not evaluate in any real validation experiment. In addition, it is not clear if there is a possibility to perceive whether the neuron output is dependent on the internal state or not. Second is the perceptron described in [44], which is a one-iteration perceptron algorithm based on unitary weights.

Despite the fact that there has been a significant amount of research involving the use of quantum properties in neural computations, the authors believe that still there is much space to improve. For example, even a precise definition of a quantum neural network that integrates neural computation and quantum computation is a nontrivial open problem [39], even though some problems in the machine learning domain may be solved efficiently on quantum computers in terms of speed, memory requirements [47], knowledge discovery, and pattern recognition and classification [46].

## 4. The Autonomous Perceptron Model

In this section, we propose a novel autonomous perceptron model that replaces the classical perceptron. The proposed APM model is able to learn the features autonomously for pattern classification with a fixed network structure. In addition, it can be implemented on a classical computer. Firstly, we define a new computational subspace that will allow the computations to run efficiently on classical computers.

### 4.1. The Computational Subspace of APM

In this section, we define a new computational subspace *M* in terms of quantum computing to suit the proposed model. The base vectors of this subspace are:

$$\left\{|0\rangle =\left(\begin{array}{c} 1\\ 0 \end{array}\right),|1\rangle =\left(\begin{array}{c} 0\\ 1 \end{array}\right)\right\}$$

Then, following the Dirac notation [26], the vector $|x_{i}\rangle \in M$ can be written as:

(3) $$|x_{i}\rangle =\alpha_{i}|1\rangle +\beta_{i}|0\rangle =\left(\begin{array}{c} \alpha_{i}\\ \beta_{i} \end{array}\right),\;|\alpha_{i}{|}^{2}+{\left|\beta_{i}\right|}^{2}=1$$

where $\alpha_{i}$ and $\beta_{i}$ are complex numbers. Therefore, we call *M* a vector subspace of the complex vector space $C^{2}$, where its bases are inspired by the mathematical representation of the qubit [26]. In this subspace, we can define two operations: addition and multiplication by scalars, as follows [48]:

$$\left(\begin{array}{c} \alpha_{i}+\alpha_{k}\\ \beta_{i}+\beta_{k} \end{array}\right)=\left(\begin{array}{c} {\tilde{\alpha }}_{i}\\ {\tilde{\beta }}_{i} \end{array}\right)$$

$$k|x_{j}\rangle =\left(\begin{array}{c} k\alpha_{i}\\ k\beta_{i} \end{array}\right)=\left(\begin{array}{c} {\tilde{\alpha }}_{j}\\ {\tilde{\beta }}_{j} \end{array}\right)$$

where k∈R and $|{\tilde{\alpha }}_{j}{|}^{2}+{\left|{\tilde{\beta }}_{j}\right|}^{2}$ does not need to equal one.

### 4.2. The Topological Structure of APM

**Figure 1:**
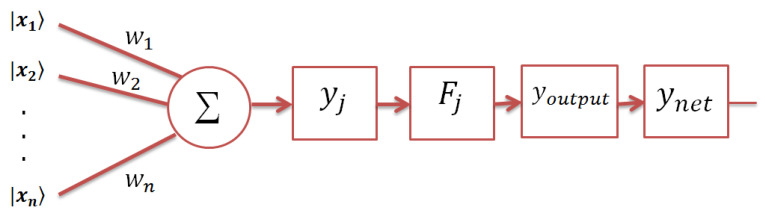
Architecture of the autonomous perceptron model (APM) model. Each input is presented as a two-dimensional normalized vector that is weighted by an operator, and then, the weighted sum $y_{j}$ is computed. Then, the activation operator $F_{j}$ for each training pattern is calculated. When a testing pattern is presented to the APM model, each activation operator is affected by the weighted sum of the test pattern. Only one activation operator is chosen, which produces the final response through the $y_{net}$ of the model.

The topological structure of the proposed model consists of a single neuron only with *n* input vectors $|x_{1}\rangle$, $|x_{2}\rangle$, …, $|x_{n}\rangle$∈M, where *n* is the dimension of the input patterns (e.g., the number of attributes). Here, the input vectors are written using the ket notation, |.〉, since they are normalized vectors and are defined according to Equation (3). A set of linear weight operators $w_{i}$ of size 2×2, where *i* = 1, 2, …, *n*, are associated with each input, and $y_{net}$ represents the final network response (see Figure 1).

Given a set of *N* training samples $\left\{(u_{j},|d_{j}\rangle )\right\}$ where j=1,2,…,N, the $j^{\mathrm{th}}$ training example is $u_{j}\in M^{n}$, e.g., $u_{j}=[|x_{j1}\rangle ,|x_{j2}\rangle ,\ldots ,|x_{jn}{\rangle ]}^{T}$, and the corresponding target is $|d_{j}\rangle =\left(\begin{array}{c} \alpha_{dj}\\ \beta_{dj} \end{array}\right)\in M$. The target vector $|d_{j}\rangle$ represents the class label of the training pattern $u_{j}$. When an input pattern $u_{j}$ is presented to the network, the weighted sum vector $y_{j}$ will be a mapping from $M^{n}$ to *M* defined as:

(4) $$y_{j}=\sum_{i=1}^{n}w_{i}|x_{ji}\rangle =\left[\begin{array}{c} \mu_{j}\\ \nu_{j} \end{array}\right],\;j=1,2,\ldots ,N$$

where $\mu_{j}$ and $\nu_{j}$ are the coefficients of the weighted sum vector of the $j^{\mathrm{th}}$ pattern in the training set. Then, the activation operator $F_{j}$ for the weighted sum $y_{j}$ is defined as:

(5) $$F_{j}=\left[\begin{array}{cc} \cos\theta_{j} & -\sin\theta_{j}\\ \sin\phi_{j} & \cos\phi_{j} \end{array}\right]$$

where j=1,2,3,…,N, and the parameters $\theta_{j}$ and $\varphi_{j}$ are two valued angles. The activation operator $F_{j}$ is a linear transformation that transforms $y_{j}$ from *M* to *M* and is defined by:

(6) $$\left[\begin{array}{cc} \cos\theta_{j} & -\sin\theta_{j}\\ \sin\phi_{j} & \cos\phi_{j} \end{array}\right]\left[\begin{array}{c} \mu_{j}\\ \nu_{j} \end{array}\right]=\left[\begin{array}{c} \alpha_{d_{j}}\\ \beta_{d_{j}} \end{array}\right]$$

where [$\mu_{j},\nu_{j}{]}^{T}$ is the weighted sum vector of the input pattern $u_{j}$, calculated by Equation (4), and $(\alpha_{d_{j}}$, $\beta_{d_{j}}{)}^{T}$ is the corresponding target vector. Each activation operator aims to transform the weighted sum vector $y_{j}$ into the given target $|d_{j}\rangle$. The weight operators are updated at time *t* using the following rule:

(7) $$w_{i}\left(t+1\right)=w_{i}\left(t\right)+\delta \;e\;\langle x_{ji}|$$

where δ is the learning rate, 0<δ≤1, $e=(y_{j}-|d_{j}\rangle )$ is the perceptron vector error and $e\langle x_{ji}|$ denotes the outer product of vectors *e* and $\langle x_{ji}|$. As the proposed model has been designed as a neural-base model that is implemented on a classical computer, the weight operator might not be a unitary operator.

The training phase corresponds to the computation of the activation operators for each pattern in the input data. For each arbitrary test pattern $u_{t}=[|x_{t1}\rangle ,|x_{t2}\rangle ,\ldots ,|x_{tn}{\rangle ]}^{T}$, the testing phase will run via two consecutive operations: First, the weighted sum is calculated through $y_{test}=\sum_{i=1}^{n}w_{i}|x_{ti}\rangle$. Second, each activation operator is applied to calculate $y_{test}$ in the form:

$$y_{output}\left(j\right)=F_{j}\;y_{test}$$

where j=1,2,…,N. These two steps can be summarized as follows:

(8) $$y_{output}\left(j\right)=F_{j}\sum_{i=1}^{n}w_{i}|x_{ti}\rangle =\left[\begin{array}{c} \alpha_{F_{j}}\\ \beta_{F_{j}} \end{array}\right]$$

Here, $y_{output}$ is a vector of *N* elements, where each element is a vector of the 2×1 vector of the network output. Equation (8) illustrates the effect of the activation operators on the weighted sum vector when a test pattern is presented to the network. Only one vector will show a response that can be specified using the following nonlinear rule:

(9) $$y_{net}=L\left(|(\left(y_{output}^{T}\circ y_{output}\right)-Z)|\right)\circ D$$

where $Z_{N\times 1}={\left[1,1,\ldots ,1\right]}^{T}$ and $D_{N\times 1}={\left[|d_{1}\rangle ,|d_{2}\rangle ,\ldots ,|d_{N}\rangle \right]}^{T}$ are the target vectors. The nonlinear function *L* retains the smallest absolute value and sets it to one and the rest of the values to zero. The operation “∘” is the Hadamard product operation [49]. We used “∘” in Equation (9), since it is clear from Equation (8) that $y_{output}$ is a vector of *N* elements, where each element is a vector of the 2×1 vector of the network output, while $y_{output}^{T}$ is the transpose of the vector $y_{output}$ such that each element of the vector $y_{output}$ is transposed. The term $\left(y_{output}^{T}\circ y_{output}\right)$ in Equation (9) applies the Hadamard product between each element of the vector $y_{output}^{T}$ and each element of the vector $y_{output}$).

To explain the Hadamard product, assume any two matrices, *A* and *B* of the same dimension m×n, then the Hadamard product A∘B is a matrix of the same dimension, as the operands, and is given by:

$${\left(A\circ B\right)}_{i,j}={\left(A\right)}_{i,j}\dot{(}{B)}_{i,j}$$

Therefore, the Hadamard product defines a binary operation that takes two matrices of the same dimensions and produces another matrix where each element (*i*, *j*) is the product of elements (*i*, *j*) of the original two matrices. For example, let $A={\left[a_{1},a_{2}\right]}^{T}$ and $B={\left[b_{1},b_{2}\right]}^{T}$. Then, the Hadamard gate in between can be given as:

$$A\circ B=\left[\begin{array}{c} a_{1}b_{1}\\ a_{2}b_{2} \end{array}\right]$$

### 4.3. The Learning Algorithm of APM

The learning algorithm of APM is composed of two phases. First is the training phase (see Algorithm 1), which embeds Equations (4), (6) and (7). In these three equations, the APM model collects information about the problem by constructing a set of activation operators. Once the training phase is finished, the second phase of APM, the testing phase is launched (see Algorithm 2). In this phase, the APM model makes a decision regarding the classification of unseen data, according to Equation (9) based on the previously collected information in the training phase. In other words, the output of the perceptron is given as a superposition of vectors given in Equation (8), whereas the final response of the perceptron is given by Equation (9). The parameters of the proposed perceptron are the following: the initial weights, the number of training examples *N*, and the learning rate δ. The best values of these parameters can be obtained through the validation dataset.

**Algorithm 1:** The training algorithm of APM.**Step 1**: Set all $F_{i}=I$ (identity matrix). Then, choose the initial weight operators $w_{i}$ randomly and the number of iterations (itr). **Step 2**: For *k* = 1 : itr            For training patterns $u_{j},j=1,\ldots ,N$            Calculate the weighted sum vector using Equation (4) for each pattern $u_{j}$.            End For            Update the weight operators using Equation (7).            End For **Step 3**: Compare each weighted sum vector for the patterns of each class label in the dataset with all other weighted sum vectors for other classes in the same dataset. In this case, it may be possible that any weighted sum vector for any class equals the weighted sum of any other class. If this is the case, then go to Step 4; otherwise, go to Step 5. **Step 4**: Re-initialize weight operators $w_{i}$ by the same way shown in Step 1, and go to Step 2. **Step 5**: Calculate the activation operator for each weighted sum qubit using Equation (6). 

When a test pattern $u_{t}=[|x_{t1}\rangle ,|x_{t2}\rangle ,\ldots ,|x_{tn}{\rangle ]}^{T}$ is presented to the APM model, the following algorithm is applied to obtain the corresponding class label.

**Algorithm 2:** The testing algorithm of APM.**Step 1**: For a test pattern $u_{t}=[|x_{t1}\rangle ,|x_{t2}\rangle ,\ldots ,|x_{tn}{\rangle ]}^{T}$ do {            Compute the weighted sum of the test pattern as:             $y_{test}=\sum_{i=1}^{n}w_{i}|x_{ti}\rangle$            For j=1:N            $y_{output}\left(j\right)=F_{j}\sum_{i=1}^{n}w_{i}|x_{ti}\rangle$
            End For            End For **Step 2**: The predicted class label of the test pattern $u_{t}$ is given by the net response of the APM as given by Equation (9).

## 5. The Computational Capability of APM

In this section, we introduce a theoretical analysis of the computational capability of the APM model. As depicted in Figure 1, the APM may have *n* inputs of the normalized vectors $\left\{|x_{i}\rangle \right\}$, i=1,2,…,n, where the attributes of each vector $\left\{|x_{i}\rangle \right\}$ are represented according to Equation (3). We can represent the weighted sum; that is given in Equation (4); as:

(10) $$y_{j}=\sum_{i=1}^{n}w_{i}|x_{ji}\rangle =\sum_{i=1}^{n}\left[\begin{array}{cc} w_{1i} & w_{3i}\\ w_{2i} & w_{4i} \end{array}\right]\left[\begin{array}{c} \alpha_{ji}\\ \beta_{ji} \end{array}\right]=\left[\begin{array}{c} \sum_{i=1}^{n}w_{1i}\alpha_{ji}+\sum_{i=1}^{n}w_{3i}\beta_{ji}\\ \sum_{i=1}^{n}w_{2i}\alpha_{ji}+\sum_{i=1}^{n}w_{4i}\beta_{ji} \end{array}\right]=\left[\begin{array}{c} \mu_{j}\\ \nu_{j} \end{array}\right].$$

Again, note that the weighted sum of the output vector $y_{j}$ is not normalized. Consequently, from Equations (8) and (10), the output $y_{output}$ can be written as:

(11) $$y_{output}\left(j\right)=F_{j}\sum_{i=1}^{n}w_{i}|x_{ti}\rangle =F_{j}\left[\begin{array}{c} \sum_{i=1}^{n}w_{1i}\alpha_{ji}+\sum_{i=1}^{n}w_{3i}\beta_{ji}\\ \sum_{i=1}^{n}w_{2i}\alpha_{ji}+\sum_{i=1}^{n}w_{4i}\beta_{ji} \end{array}\right]$$

Then, the final output of APM is determined by applying the nonlinear function *L* according to Equation (9). Indeed, this function was constructed based on a single activation operator *F* among the set of activation operators $F_{j}$, where j=1,2,…,N. Accordingly, the final output of the APM model can be represented as:

(12) $$y_{net}=F\left[\begin{array}{c} \sum_{i=1}^{n}w_{1i}\alpha_{ji}+\sum_{i=1}^{n}w_{3i}\beta_{ji}\\ \sum_{i=1}^{n}w_{2i}\alpha_{ji}+\sum_{i=1}^{n}w_{4i}\beta_{ji} \end{array}\right]$$

**Figure 2:**
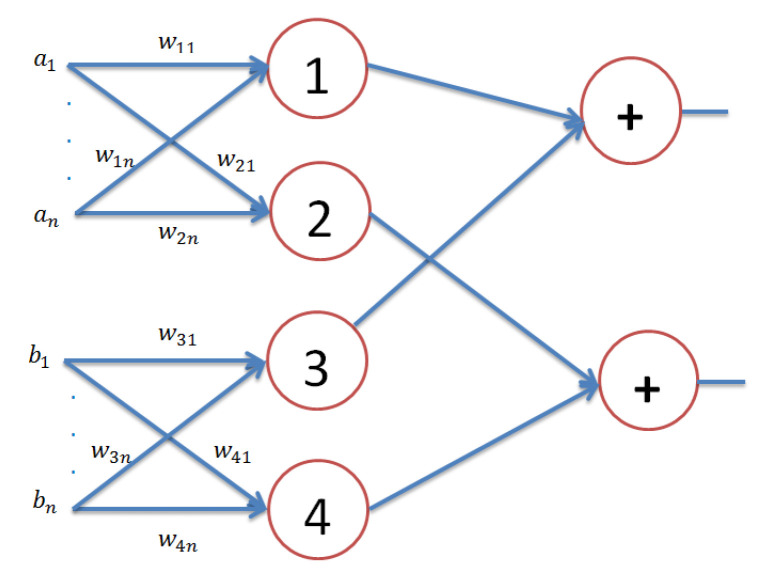
A classical three-layer feed-forward neural network has 2n neurons in the input layer, 4 neurons in the hidden layer, and 2 neurons in the output layer.

On the other hand, if we assume that we have a classical neural network with three layers with 2n neurons in the input layer, four neurons in the hidden layer, and two neurons in the output layer, as depicted in Figure 2, the output $O_{t}$ of this network will be:

(13) $$O_{t}=\left[\begin{array}{c} O_{t_{1}}\\ O_{t_{2}} \end{array}\right]=\left[\begin{array}{c} f\left(\sum_{i=1}^{n}w_{1i}\alpha_{ji}\right)+f\left(\sum_{i=1}^{n}w_{3i}\beta_{ji}\right)\\ f\left(\sum_{i=1}^{n}w_{2i}\alpha_{ji}\right)+f\left(\sum_{i=1}^{n}w_{4i}\beta_{ji}\right) \end{array}\right]$$

by considering that F=I and f(x)=x in Equations (12) and (13), respectively. Simply, although the APM model has only a single neuron, it possesses the same computational power of three layers of the feed-forward neural network with the neuron topology 2n−4−2, where *n* is the number of neurons. On the other hand, *F* is a nonlinear operator in Equation (12), as depicted in Equation (5). It is clear that the APM model that has only a single neuron performs the same nonlinear mapping that can be performed by the three-layer classical neural network.

## 6. Experiments and Results

To demonstrate the robustness, efficiency and effectiveness of our model, we evaluated the performance of the proposed APM model in two different ways. First, we evaluated the performance of the proposed model in learning a particular logical function, when compared with other quantum-based perceptron models. Second, we show the performance of our model in a number of classification applications and compare it with different parametric and non-parametric classification models.

### 6.1. Learning a Logical Function

To illustrate the autonomous learning of a particular example of logical functions, such as the XOR function, we assumed that there was a set of training data pairs $P_{i}$ = {$x_{i}$, $d_{i}$}, *i* = 1, 2, 3 and 4, where the feature vector included two features $x_{i}\in \left\{x_{i1},x_{i2}\right\}$, and the desired output d∈{+1,−1} is a binary function [20]. Then, the input patterns can be represented using the states of a two-dimensional system, i.e., a qubit, so that each pattern is given by one of the basis states $|x_{i}\rangle \in =\left\{|0\rangle ,|1\rangle \right\}$, for *i* = 1 and 2, where |0〉 and |1〉 denote the computational bases, in the *M* subspace, for each pattern, as shown in Section 4.1.

Accordingly, we can define the two classes of the XOR function, where each class includes two patterns:

**Class A:**

(14) $$\begin{array}{ccc} P_{1} & = & \left\{|x_{11}\rangle =|0\rangle ,|x_{12}\rangle =|0\rangle ,|d_{1}\rangle =|0\rangle \right\}\\ P_{2} & = & \left\{|x_{21}\rangle =|1\rangle ,|x_{22}\rangle =|1\rangle ,|d_{2}\rangle =|0\rangle \right\} \end{array}$$

**Class B:**

(15) $$\begin{array}{ccc} P_{3} & = & \left\{|x_{31}\rangle =|0\rangle ,|x_{32}\rangle =|1\rangle ,|d_{3}\rangle =|1\rangle \right\}\\ P_{4} & = & \left\{|x_{41}\rangle =|1\rangle ,|x_{42}\rangle =|0\rangle ,|d_{4}\rangle =|1\rangle \right\} \end{array}$$

Furthermore, assume that the weight operators are randomly initialized as $w_{1}=w_{2}=\left[\begin{array}{cc} 1.1 & 1.2\\ 0 & 0 \end{array}\right]$.

The learning procedure of the XOR function can be explained as follows: When the four patterns are presented as input to the APM model, the weighted sum of each pattern is:

(16) $$y_{1}=\left[\begin{array}{c} 2.2\\ 0 \end{array}\right],\;y_{2}=\left[\begin{array}{c} 2.4\\ 0 \end{array}\right],\;y_{3}=y_{4}=\left[\begin{array}{c} 2.3\\ 0 \end{array}\right]$$

Hence, $y_{3}=y_{4}$, since the two patterns $P_{3}$ and $P_{4}$ are in the same class, i.e., Class B, and their values are exchanged (see Equation (15)). Since the first and second patterns have different values (see Equation (14)), then each of the third and fourth weighted sum values, i.e., $y_{1}$ or $y_{2}$, will have different values. Therefore, as we have only three weighted sum variables, this implies that we have only three activation operators, where $F_{3}=F_{4}$ will take the same value based on the initial values of $w_{1}$ and $w_{2}$. The three activation operators are:

$$F_{1}=\left[\begin{array}{cc} 0.4545 & -0.8907\\ 0 & 1 \end{array}\right],\;F_{2}=\left[\begin{array}{cc} 0.4167 & -0.9091\\ 0 & 1 \end{array}\right],$$

(17) $$F_{3}=\left[\begin{array}{cc} 0 & -1\\ 0.4348 & 0.9005 \end{array}\right]$$

Then, as given in Equation (8), the superposition output can be calculated as follows:

(18) $$y_{output}\left(j\right)=F_{j}\sum_{i=1}^{n=2}w_{i}|x_{ti}\rangle$$

As pattern $P_{4}$ is not used in training, we can use it as a test pattern to the APM model. Then, the weighted sum for this pattern will be:

$$\begin{array}{c} y_{output}\left(1\right)=\left[\begin{array}{cc} 0.4545 & -0.8907\\ 0 & 1 \end{array}\right]\left[\begin{array}{c} 2.3\\ 0 \end{array}\right]=\left[\begin{array}{c} 1.0454\\ 0 \end{array}\right],\\ y_{output}\left(2\right)=\left[\begin{array}{cc} 0.4167 & -0.9091\\ 0 & 1 \end{array}\right]\left[\begin{array}{c} 2.3\\ 0 \end{array}\right]=\left[\begin{array}{c} 0.9584\\ 0 \end{array}\right],\\ y_{output}\left(3\right)=\left[\begin{array}{cc} 0 & -1\\ 0.4348 & 0.9005 \end{array}\right]\left[\begin{array}{c} 2.3\\ 0 \end{array}\right]=\left[\begin{array}{c} 0\\ 1 \end{array}\right] \end{array}$$

Therefore, $y_{output}$ is given as follows:

(19) $$\begin{array}{c} y_{output}={\left[\left[\begin{array}{c} 1.0454\\ 0 \end{array}\right],\left[\begin{array}{c} 0.9584\\ 0 \end{array}\right],\left[\begin{array}{c} 0\\ 1 \end{array}\right]\right]}^{T} \end{array}$$

After calculations, Equation (9) is used to decide which vector in Equation (19) will be the output of the APM network for the current test pattern, as follows:

$$\begin{array}{ccc} y_{net} & = & L\left(|(\left[1.093\;\;0.919\;\;1.000\right]-{\left[1\;1\;1\right]}^{T})|\right)\circ D\\ & = & {\left[0\;0\;1\right]}^{T}\circ {\left[\left[1\;0\right]\;\left[1\;0\right]\;\left[0\;1\right]\right]}^{T}={\left[0\;1\right]}^{T}=|1\rangle \end{array}$$

Then, the output of pattern $P_{4}$ is |1〉, which is correct, as given in Equation (15).

Note that the proposed APM model requires only three patterns in order to learn the XOR logical function and gives a response after only one iteration. Table 1 shows a comparison of the learning capabilities of the proposed APM model and other quantum-inspired perceptron counterparts [16,20]. Note that the new proposal for a perceptron requires less patterns and iterations to learn the XOR function when compared with the quantum perceptron (QP) [16], which requires four input patterns and 16 iterations to fulfil the learning. Similar to APM, the autonomous quantum perceptron (AQP) [20] requires only one iteration to give a response; however, it shares the same limitations as QP in terms of the requirement of the four input patterns to learn the XOR function autonomously. Overall, the proposed autonomous perceptron method is more efficient and requires less computation resources than both the classical and quantum counterparts in learning the logical XOR function.

### 6.2. Classification Experiments

For a fair assessment, we conducted the classification experiments using both real datasets and artificial datasets using our model and other state-of-the-art models.

#### 6.2.1. Experimental Settings

Four benchmark real datasets were used in this comparison, namely the IRIS dataset, the Breast Cancer dataset, the Types of Glass dataset, and the Wine Vintage dataset. These datasets were downloaded from the website of the University of California Irvine (UCI) Machine Learning Repository [50]. For each dataset, we randomly divided the dataset into 50% for the training phase, 15% for the validation phase, and 35% for the testing phase. In all experiments, we unified the following conditions: First, we used the lowest limit of training data samples (random selection among 50%) that was enough to show better performance by all models. Second, the testing data were selected to be completely unseen previously in the training. Third, the validation data were used to fine-tune the parameters of all models, and then, we fixed all parameters accordingly during the testing phase. Table 2 shows the features of each dataset adopted in our experiments.

In these experiments, we compared the performance of the proposed model with 15 reference models. Most of these models were key selections in machine learning and data mining communities to achieve different pattern classification applications. In each model, if any model selection was required, then we exploited the conventional optimal model selection for the corresponding model. To unify the implementation medium of all models, all experiments shown in this paper were conducted on a desktop PC with a CPU configuration as 3.5 GHz Intel(R) Xeon(R) E5-1620 v3, and 32.00 GB RAM on a 64-bit OS operating system. The experiments of the proposed model and the reference models were implemented using MATLAB Toolbox Release R2016a, The MathWorks, Inc., Natick, MA, USA.

#### 6.2.2. Experimental Results

In the following section, we summarize the experimental results obtained by the proposed model and the reference models to solve pattern classification problems using real and synthetic datasets.

A. Classification of real dataset

Table 3 illustrates a comparison of the number of neurons required by the proposed perceptron and the multilayer perceptron approaches to perform the highest classification rate. In addition, Table 4 shows the classification accuracy of the proposed model and reference models.

As shown in Table 3 and Table 4, the proposed APM outperformed other models. This is due to two significant properties: First, in contrast with the multilayer perceptron neural network, the APM had a fixed network structure with only one neuron to classify nonlinear classification problems, as shown in Table 3. Second, the APM outperformed the reference models in terms of classification accuracy, especially in the first three datasets, as shown in Table 4. This table shows that the best model among the reference models was the subspace model. Table 5 shows the parameters of the subspace model to classify the datasets at hand.

It is easy to notice in Table 4 that some baseline models showed lower performances than expected. For example, in the Breast Cancer experiment, the random forest, multilayer perceptron, and naive Bayes models showed classification accuracies of 30.4, 48.4 and 64.3, respectively. This is not normal behaviour for these standard models in classification problems. Nevertheless, in order to understand these results, it is worth mentioning that the most important aspect of the proposed model was the limited number of training samples *N* that were needed to train the model. This number *N* was decided using validation dataset samples. According to Table 2, we used 1.48% of the whole Breast Cancer dataset to train the APM. To give a fair comparison in this experiment, this portion of the dataset was used to train all models. Of course, this portion of training data was very low for training baseline models such as random forest, multilayer perceptron, and naive Bayes. Certainly, the performance of these models would be improve drastically if we increased the number of training samples. Therefore, it would be worth comparing not only the performance, but also the rate of training data necessary to achieve better results.

B. Classification of synthetic dataset 

It is known that synthetic data are computer-generated data that mimic real data, but are less expensive and more easily labelled than real data. Here, we continue to investigate the robustness of the proposed APM approach using a classification problem of two-overlapped classes using synthetic data. This type of classification problem can be interpreted as a complex generalization of the XOR problem. This classification problem is usually treated in the literature using only nine training patterns [15,51].

**Figure 3:**
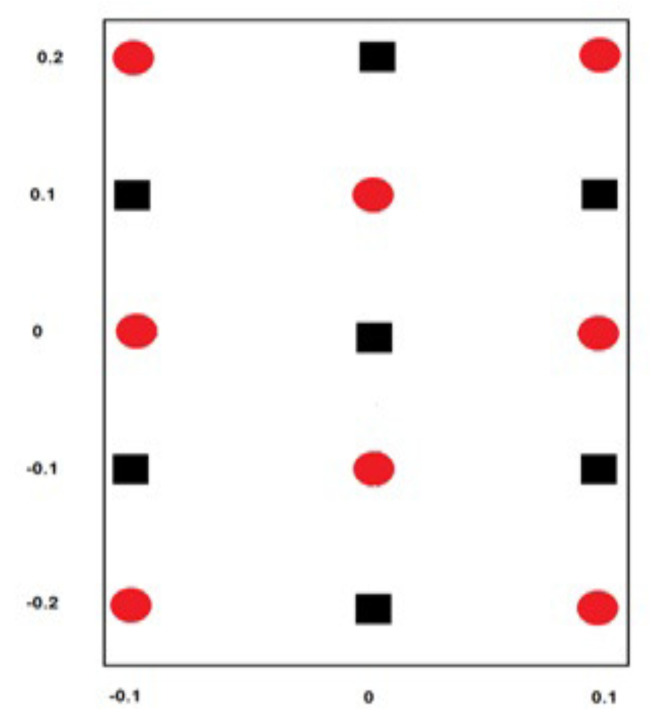
A classification problem of two overlapping classes.

However, to ensure a robust assessment of the APM model, we treated this problem using 15 training patterns and 176 testing patterns (for simplicity, a simulation of the 15 training patterns is shown in Figure 3). As depicted in Figure 3, the training patterns comprised two distinguished overlapping classes: the first was the oval-shaped class, assigned a target |0〉, while the second was the square-shaped class, assigned a target |1〉 with arbitrary input patterns, shown in Table 6 and Table 7, where the values of the input patterns are classical data. For convenience, it is preferred to write these values as vectors in *M* space in the form |η〉=a|0〉+b|1〉, where ${\left|a\right|}^{2}+{\left|b\right|}^{2}=1$. For example, the classical pattern $P_{1}=\left(0.1,0\right)$ can be written in *M* space as:

(20) $$P_{1}=\left\{|x_{1}\rangle =\left[\begin{array}{c} 0.1\\ 0.9950 \end{array}\right],|d_{1}\rangle =\left[\begin{array}{c} 1\\ 0 \end{array}\right]\right\}.$$

**Figure 4:**
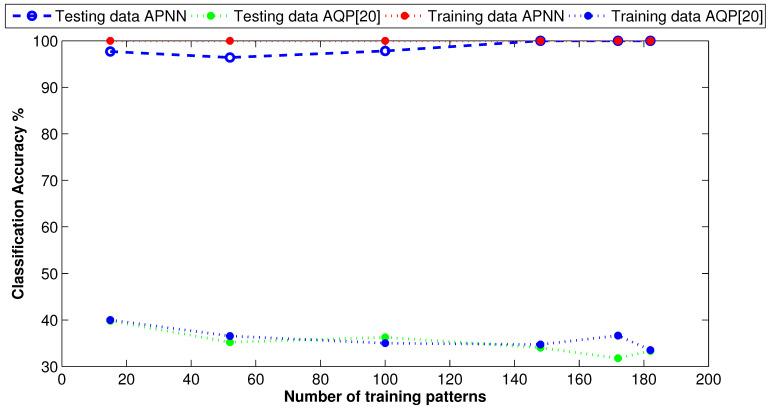
Classification curve of two overlapped classes using synthetic data.

For performance evaluation, we compared the APM model with the autonomous quantum perceptron (AQP) [20]. Figure 4 demonstrates the performance of both approaches in the training and testing phases. As shown in Figure 4, in the training phase, the proposed approach achieved a classification accuracy of 100%. In contrast, the AQP approach [20] achieved, at most, 40% as a classification accuracy. In the testing phase, the APM approach started with 98% accuracy, but as we increased the number of testing patterns, the accuracy increased to 100%. At the same time, the AQP approach [20] still performed the worst even when we increased the number of testing patterns.

## 7. Time Complexity Analysis

The time complexity of any algorithm is defined as the amount of time taken by the algorithm to run as a function of the number of inputs [52]. However, the computational time required to train a neural network is affected by many factors such as the number of neurons, the dimension of the features, the number of training samples, and the number of epochs required for the training phase to converge [52]. In pattern classification problems, the number of classes in the dataset is related to the number of neurons in the neural networks and, sometimes, to the number of output neurons. In the following section, we analyse the time complexity of the proposed model along with that of the baseline models.

### 7.1. Comparison with Baseline Classifiers

During the assessment experiments that yielded the results provided in Table 4, we also wrote down the implementation time, in seconds, for all models. To ensure a fair assessment, we unified all experimental settings for all models. We ran each model separately using the given dataset and wrote down the CPU time that was calculated automatically by the MATLAB function “*cputime*”. The results are shown in Table 8, where it is clear that the CPU time required by the proposed model was much less than the time required by all other reference models.

### 7.2. Comparison with Multilayer Perceptron MLP using Big-O Analysis

#### 7.2.1. Big-O Analysis for the MLP Model

Here, we compare the time complexity of the proposed APM model with that of the multilayer perceptron MLP [53] model using asymptotic notations, namely big-O analysis. From the experimental point of view, conducting an accurate time complexity analysis of two neural network algorithms with different architectures is a very labour-intensive, as well as unfair process, since time complexity is machine dependent. In such a case, the simplest way to compare the time complexity of two algorithms is by calculating the maximum number of primitive (or dominant) operations that each algorithm executes to converge [52], which can be represented widely using the big-O notation. Intuitively, the number of dominant operations depends on the amount of input data.

It was demonstrated in [52] that the time complexity for training a neural network can be expressed in terms of the number of input nodes *n*, the number of hidden nodes *H*, the number of output nodes *o*, and the number of training samples, *N*. It can take the following form [52]:

(21) $$\Gamma^{nodes}\left(n,H,o,N\right)=\left[3\times n\times H+4\times o\times \left(H+1\right)+3\times H\right]\times N$$

If we consider the number of epochs to be *P*, which is required to calculate the algorithm’s convergence during the training phase, then Equation (21) takes the form:

(22) $$\Gamma^{nodes}\left(n,H,o,N,P\right)=\left(\left[3\times n\times H+4\times o\times \left(H+1\right)+3\times H\right]\times N\right)\times P$$

To ensure a fair comparison, we consider that both the number of input nodes *n* and the number of training samples *N* are constant for the same dataset, but using different classifiers. Then, we can represent this formula using big-O analysis for the MLP model as:

(23) $$\Gamma^{nodes}\left(n,H,o,N,P\right)=\left(\left[3\times n\times H+4\times o\times \left(H+1\right)+3\times H\right]\times N\right)\times P=O\left(H,o,P\right)$$

#### 7.2.2. Big-O Analysis for the APM Model

For the APM model, assume that $w^{APM}$ is the total number of weights used in this model. Then, complexity can be defined as:

$$w^{APM}=4\times n$$

where *n* is the number of dimensions of training patterns. Each training pattern introduced to the APM model requires an activation operator, which is given in Equation (5). Then, each training pattern requires four multiplications, which are included in Equation (6), and another four multiplications to solve this equation, as well. As we explained before, the APM has a fixed structure that includes only one neuron, i.e., no hidden or output neurons. Thus, for *N* training samples, the time complexity of the APM training phase is given as follows:

(24) $$\begin{array}{cc} \Gamma^{APM}\left(n,N,P\right) & =(([4\times n+4+4]\times N)\times P)\\ & =([4n+8]\times N)\times p \end{array}$$

Again, if we consider that both the number of input nodes *n* and the number of training examples *N* are constant for the same dataset for different classifiers, then the big-O analysis of the APM model will be given as:

(25) $$\begin{array}{c} \Gamma^{APM}\left(n,N,P\right)=\left(\left[4n+8\right]\times N\right)\times p=O\left(P\right) \end{array}$$

From Equations (23) and (25), it is clear that the time complexity of the MLP model depends on the number of hidden nodes *H*, the number of output nodes *o*, and the number of epochs *P*. In contrast, the time complexity of the APM model depends only on the number of epochs for the same dataset. This implies that the proposed APM model has a time speedup compared with the MLP.

## 8. Results’ Discussion and Analysis

We present now an overview and discussion of the main contributions and findings of this paper. In recent years, the remarkable importance of machine learning has led to a host of studies that have tried to bridge between quantum computing and machine learning. There have been several attempts to develop quantum-inspired perceptron models. In this paper, we proposed a novel autonomous perceptron model (APM) inspired by the definition of the qubit. The proposed perceptron has an optimal neural structure of only one single neuron to classify nonlinear separable datasets using a limited number of training samples.

As a nonlinear model, the APM is able to autonomously and efficiently construct the activation operators, even in complex feature space problems. In this paper, we showed that the proposed perceptron, with one neuron, has the same computational power of a classical neural network of three layers with the neuron topology 2n−4−2, where *n* is the number of neurons. We validated the APM model in different classification experiments using different datasets, where it showed better performance than its baseline counterparts.

First, we used the APM to learn the logical XOR function. Table 1 shows that the training requirements of APM to learn the XOR function are simpler than its counterparts [16,20], as well as multilayer perceptron. Not only the logical XOR function, but also we investigated; in the early draft of this paper [54], the learning of other logical gates, such as Hadamard-gate and Not-gate, where we found that APM showed better performance compared to other models. Moreover, classical perceptron, as a linear model, is incapable of learning nonlinear functions using a single neuron [20,55].

Second, we validated the proposed APM model to perform several classification applications using four real datasets, namely IRIS, Breast Cancer, Types of Glass and Wine Vintage. Each dataset includes a different number of classes, attributes and input patterns, as shown in Table 2. The proposed APM outperformed 15 standard classification models across the same experimental conditions. As shown in Table 4, the APM accuracy was optimum and approximately reached the highest accuracy. In contrast, the multilayer perceptron (MLP) showed worse performance for datasets with two classes and improved slightly when the dataset had more than two classes. The same observation was noticed in the performance of other baseline classifiers listed in Table 4 where the performance was sensitive to the number of classes and attributes. This gives us an indication that these classifiers were data-dependent, unlike APM, which performed ideally in all situations.

**Figure 5:**
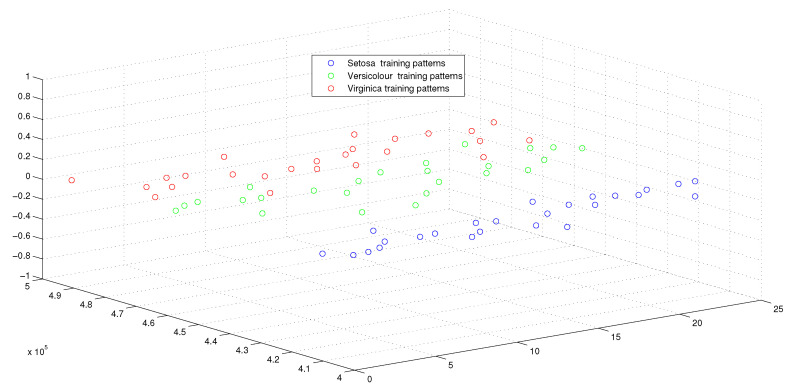
The weighted sum of training patterns of the IRIS dataset.

**Figure 6:**
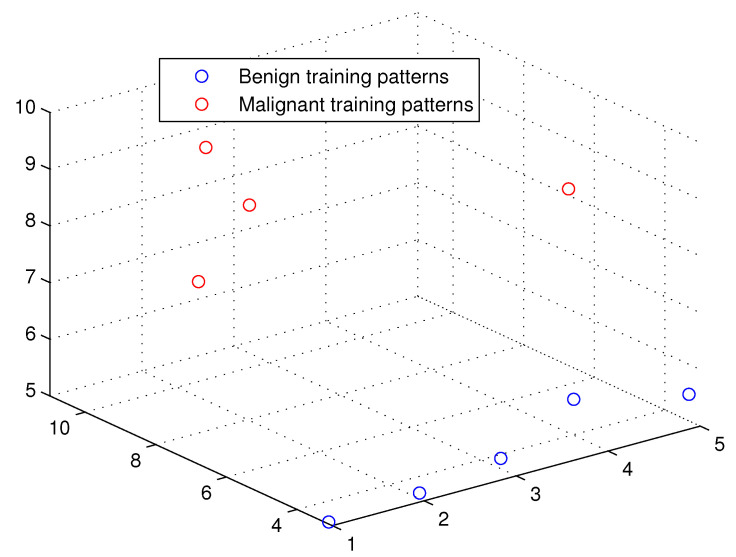
The weighted sum of the training patterns of the Breast Cancer dataset.

**Figure 7:**
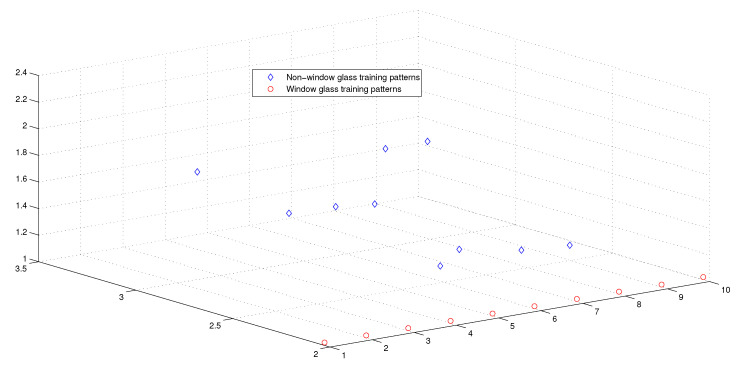
The weighted sum of the training patterns of the Types of Glass dataset.

**Figure 8:**
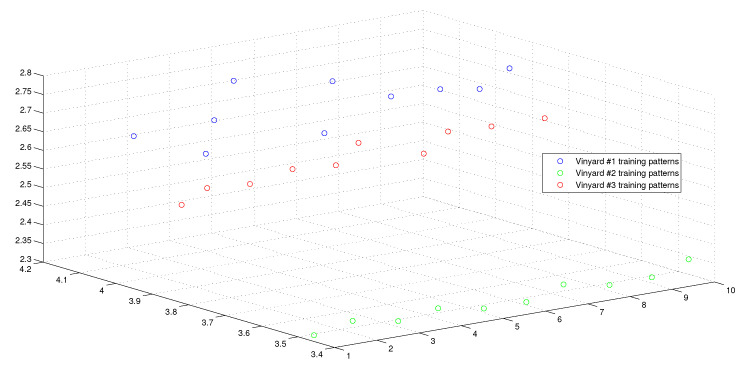
The weighted sum of the training patterns of the Wine Vintage dataset.

This optimum performance of APM compared with the reference models was due to three factors: (i) As we showed in Section 5, the proposed perceptron with one neuron had the same computational power of a classical neural network of three layers with the neuron topology 2n−4−2, where *n* is the number of inputs. (ii) As a result of representing each input vector into a two-dimensional vector and weighting it with an operator, it was implied that the proposed model maximized the separability between the patterns in the weighted sum vector space, as depicted in Figure 5, Figure 6, Figure 7 and Figure 8. (iii) Although the weighted sum of the testing patterns may overlap when test patterns were fed into APM, each transfer operator $F_{j}$ was projected into the corresponding class region accurately. In other words, each training weighted sum had a transfer operator, calculated using Equation (6), to self-regularize the decision boundary between distinct classes. Therefore, the set of activation operators gave the proposed algorithm the potential to self-regulate, which ensured optimum classification results were achieved, as shown in Table 4.

**Figure 9:**
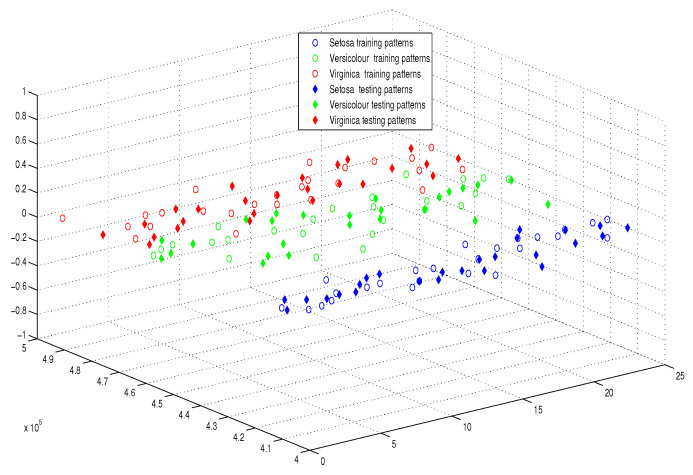
The weighted sum of the training and testing patterns of the IRIS dataset.

**Figure 10:**
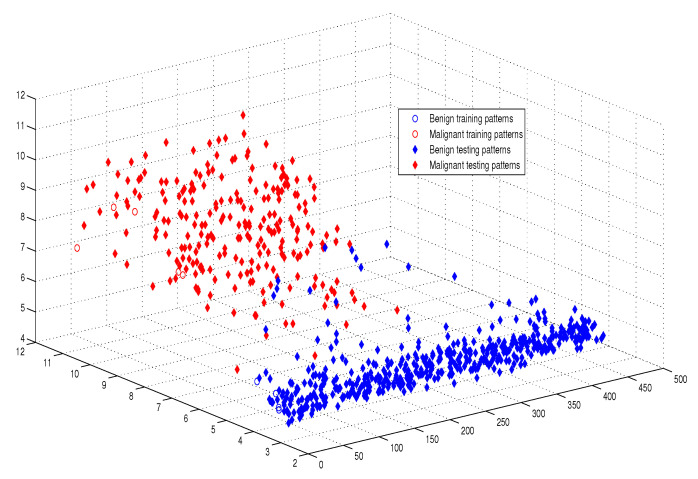
The weighted sum of the training and testing patterns of the Breast Cancer dataset.

**Figure 11:**
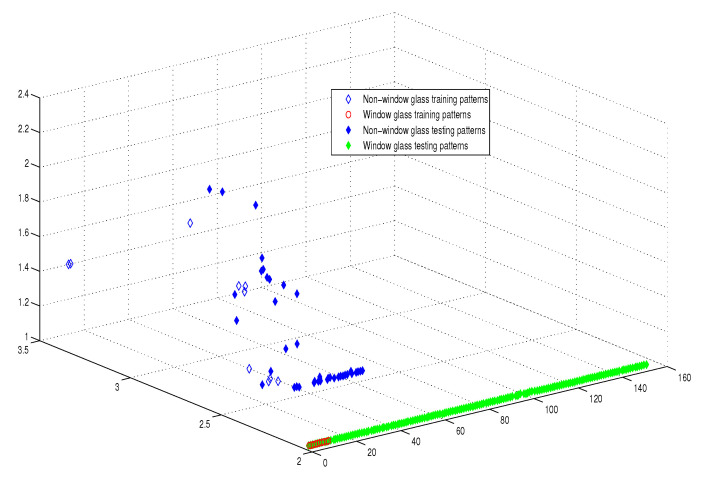
The weighted sum of the training and testing patterns of the Types of Glass dataset.

**Figure 12:**
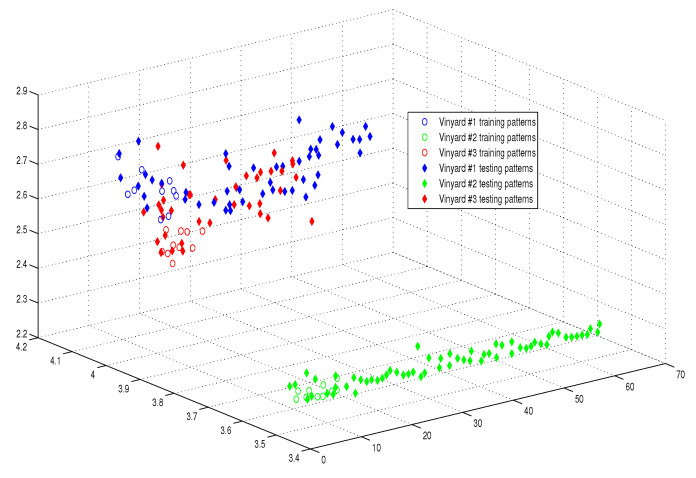
The weighted sum of the training and testing patterns of the Wine Vintage dataset.

Moreover, as illustrated in Figure 9, Figure 10, Figure 11 and Figure 12, the APM model had the potential to deal with the overlapped distribution included in the given classes, and it outperformed other models. As a result, this maximized the separability among the patterns in the weighted sum vector space due to extending each input vector into a two-dimensional vector and weighting it with an operator. This illustrates the ideal performance of the proposed model compared with the reference models, for instance, in the case of the Type of Glass experiment, as depicted clearly in Figure 7 and Figure 11. Overall, the previously mentioned properties imply that the proposed APM had a non-linear mapping with activation operators and network structures, which asserts the potential of the proposed model to classify nonlinear separable problems efficiently. Indeed, this property distinguishes the proposed algorithm from other algorithms and opens the door widely for more achievements in the quantum-inspired machine learning modelling.

Third, we conducted another classification experiment using a synthetic dataset including two overlapped classes. In this experiment, we conducted a comparison with another autonomous quantum perceptron (AQP) counterpart [20]. In the training and testing phases, we found that APM is more robust to the selection of training samples and can efficiently converge using a limited amount of training data, where classification performance was optimum, as shown Figure 3. In contrast, the AQP failed to learn a nonlinear separable function, even when the number of training patterns was increased. On the other hand, the APM demonstrated its consistency when increasing the number of training patterns.

Fourth, the validation experiment confirmed that the superiority of the APM was not exclusive to the classification accuracy or learning requirement perspectives, but also included the execution time that was required to converge. As demonstrated in Table 8, the implementation time required by the APM was much less than that for other standard classifiers under the same experimental conditions. Moreover, we showed that the APM required fewer dominant operations to converge than the number of dominant operations that were required by the MLP to converge, also under the same experimental conditions.

Overall, it is clear that the computational requirements of the proposed APM model were lower than those of its counterparts. This was due to the fixed architecture of the model, which was constructed independently of the data at hand. Furthermore, the properties of the proposed perceptron, which were inspired by the superposition power of the qubit, enabled the model to converge more quickly than its classical counterparts.

## 9. Conclusions

In recent years, the remarkable importance of machine learning has led to a host of studies that have tried to bridge between quantum computers and artificial neural networks. In this paper, we introduced a new autonomous perceptron model (APM) with a fixed architecture including only one neuron, which was inspired by the computational power of the quantum bit. Using a limited number of training samples, the APM can classify different problems of nonlinear separable classes. As a nonlinear model, the APM is capable of autonomously constructing the activation operators to achieve efficient learning, even in complex feature space problems. Experimentally, we showed that although the structure of the proposed neural model contained only one neuron, it could classify the XOR function, which cannot be classified using a single layer of traditional neurons. Furthermore, the APM model classified the XOR function using only three patterns out of four, which cannot be done using other neural models. Empirically, the superiority of the APM to perform classification using benchmark datasets was demonstrated, and the model compared favourably with both machine learning approaches and its quantum neural network counterparts. The superiority of the APM was not exclusive to classification accuracy only, but also, the execution time required for the APM to converge was less than that of its counterparts. In future works, we plan to investigate the optimum initial weights that could be used by APM to suit the problem at hand.

## Figures and Tables

**Table 1:** Comparison of learning capabilities among the proposed APM perceptron, the autonomous quantum perceptron (AQP) [20], and the quantum perceptron (QP) [16].

Name of Algorithm	APM	AQP [20]	QP [16]
No. of iterations	1	1	16
No. of training patterns	3	4	4

**Table 2:** Features of the real benchmark datasets used in the experiments.

Name of Dataset	IRIS	Breast Cancer	Types of Glass	Wine Vintage
No. of attributes	4	9	9	13
No. of classes	3	2	2	3
No. of training examples (N)	48%	1.48%	16.85%	25%
No. of patterns	150	699	214	178

**Table 3:** A comparison of the number of neurons that required for the proposed perceptron APM and the multilayer perceptron (MLP).

Name of Algorithm	IRIS	Breast Cancer	Types of Glass	Wine Vintage
The Proposed Perceptron APM	1	1	1	1
Multilayer Perceptron (MLP)	4-5-3	9-26-2	9-14-2	13-10-3

**Table 4:** The classification accuracy (%) of the proposed APM model and reference models.

Name of Algorithm	Non-Linearity Property	IRIS	Breast Cancer	Types of Glass	Wine Vintage
Proposed model APM	yes	**99.6**	**98.8**	**100**	**100**
1- Multilayer perceptron	yes	96	48.4	61.1	93.9
2- Linear discriminant	no	94.7	96.6	90.7	**100**
3- Quadratic discriminant	yes	93.3	48.4	89.7	**100**
4- LogitBoost	yes	94.7	65	62.2	95.3
5- Naive Bayes	yes	96	64.3	48.9	93.9
6- K* Classifier	yes	94.7	49.1	73.3	91.9
7- Bagging	yes	92	97.1	94.4	**100**
8- MetaClassifier	yes	94.7	48.4	55.5	95.9
9- Decision table	yes	94.7	70.7	91.6	**100**
10- Random forest	yes	94.7	30.4	77.8	91.9
11- Logistic model Trees	no	94.7	65	65.5	95.3
12- Linear SVM	no	95.3	94.1	87.9	**100**
13- Fine KNN	yes	96	94.1	93.5	**100**
14- AdaBoost	yes	33.3	91.2	76.6	37.5
15- Subspace	yes	94.7	91.2	95.3	**100**

**Table 5:** The parameters of the subspace model to classify the IRIS, Breast Cancer, Types of Glass and Wine Vintage datasets, where “NN” refers to the Nearest Neighbour model.

Name of Algorithm	IRIS	Breast Cancer	Types of Glass	Wine Vintage
Type of learner	NN	NN	NN	NN
Number of learners	30	30	30	30
Subspace Dimensions	2	7	5	7

**Table 6:** Training patterns of the oval-shaped class.

$P_{1}$	$P_{2}$	$P_{3}$	$P_{4}$	$P_{5}$	$P_{6}$	$P_{7}$	$P_{8}$
(0.1,0)	(0.1,0.2)	(0,0.1)	(−0.1,0.2)	(−0.1,0)	(0,−0.1)	(0.1,−0.2)	(−0.1,−0.2)

**Table 7:** Training patterns of the square-shaped class.

$P_{9}$	$P_{10}$	$P_{11}$	$P_{12}$	$P_{13}$	$P_{14}$	$P_{15}$
(0.1,0.1)	(0,0)	(0,0.2)	(−0.1,0.1)	(0.1,−0.1)	(−0.1,−0.1)	(0,−0.2)

**Table 8:** Time complexity comparison (in seconds) among the proposed APM classifier and other standard classifiers.

Algorithm Name	IRIS	Breast Cancer	Types of Glass	Wine Vintage
APM	0.06	0.08	0.05	0.07
Linear Discriminant	0.23	0.19	0.22	0.21
Quadratic Discriminant	0.18	1.87	0.19	0.13
Bagging	0.72	0.8	0.86	0.83
Decision Table	0.89	1.05	0.7	0.34
Linear SVM	1.14	0.7	0.26	0.35
Fine KNN	0.26	0.42	0.24	0.23
AdaBoost	0.31	1.56	0.32	0.23
Subspace	1.14	1.29	0.97	0.87
